# Non-specific Low Back Pain and Postural Control During Quiet Standing—A Systematic Review

**DOI:** 10.3389/fpsyg.2019.00586

**Published:** 2019-03-22

**Authors:** Cathrin Koch, Frank Hänsel

**Affiliations:** Department of Sport Psychology, Institute of Sport Sciences, Technische Universität Darmstadt, Darmstadt, Germany

**Keywords:** non-specific low back pain, postural control, standing, EMG, kinematics

## Abstract

**Background:** There is a great number of people who require treatment for non-specific low back pain (LBP) yet the causes are still unclear. One proposed cause for LBP is impaired motor control and more specific an impaired postural control.

**Objective:** The purpose of this review is to provide an overview of postural control parameter differences in persons with and without non-specific LBP during quite standing.

**Methods:** A literature search in five databases from January 2000 until January 2018 was performed and was followed by a hand search. Twenty-one articles comparing healthy adults and adults with non-specific LBP in neuromuscular and/or biomechanical parameters during bipedal stance without external perturbation in lab studies were examined. Data extraction and quality assessment were independently performed by two persons. Factors such as study population, outcome measures, and results were extracted from the articles and included in this analysis.

**Results:** The results show that persons with and without non-specific LBP differed in several parameters of postural control such as the center of pressure displacement, postural control strategy, and muscle activation patterns.

**Conclusion:** While the results show that none of the parameters alone lead to significant effects, the combination of neuromuscular and biomechanical parameters was associated with the impairment of postural control in individuals with LBP during standing. Since the studies included in this analysis used different methodological procedures a replication of these studies with standardized procedures is imperative for the acquisition of more conclusive evidence on the differences in postural control during standing.

## Introduction

The lifetime prevalence of low back pain (LBP) in industrial countries is at 84% (Hildebrandt et al., 2004). Approximately 85% of such back pain is classified as non-specific, which means that no structural change, no inflammation and no specific disease can be found as its cause (O'Sullivan, 2005). The number of people who require treatment for back pain is high (Saragiotto et al., 2016). The relation between altered motor control and LBP, for example in activities of daily living, has been discussed (Ruhe et al., 2011a; Laird et al., 2014; Ghamkhar and Kahlaee, 2015; Saragiotto et al., 2016). Of major interest is also the alteration of postural control, which is the ability to stabilize the trunk, due to its high prevalence in daily life.

Existing theories provide a basis for explaining altered neuromuscular activity while under pain (van Dieën et al., 2003; Hodges and Tucker, 2011). One theory proposed by Hodges and Tucker (2011) implied a redistribution of activity within and between muscles, which changes mechanical behavior and, thereby, leading to the protection from further pain and injury. However, altered postural control is not explained by simple changes in excitability of the motor system. The changes exist on multiple levels of the motor system. In the model it is postulated that changes of the nervous system in reaction to pain lead to alterations in the neuromuscular system that may have a complementary, additive, or competitive effect. Pain leads to a redistribution of activity, which in turn means that mechanical behavior changes. While these alterations come with short-time benefits, they are followed by negative consequences in the long-term (Hodges and Tucker, 2011).

Based on this theory, neuromuscular activation patterns and the effect on biomechanical outcome parameters have to be considered to evaluate postural control during standing. A requirement to ensure stability even during quiet stance is a functioning interaction between the neural and the musculoskeletal system (Shumway-Cook and Woollacott, 2007). Therefore, it is possible to gain a full impression on postural control only by considering changes on a neuromuscular and biomechanical level of postural control. Altered activity patterns of abdominal and extensor muscles of the back (Ghamkhar and Kahlaee, 2015) indicate that postural control in LBP is disrupted on a neuromuscular level. In some studies, we can see a restricted range of motion in the lumbar region on a biomechanical level (Laird et al., 2014), and a changed center of pressure (CoP) oscillations (Radebold et al., 2001).

No comprehensive review of postural control and non-specific LBP in quiet standing could be found considering both neuromuscular and biomechanical parameters. So far, there are two reviews examining differences in biomechanical parameters, however, they do not distinguish between specific and non-specific back pain (Mazaheri et al., 2013; Laird et al., 2014), even though their differentiation has been recommended in the literature (Koes et al., 2010). To exclude studies with heterogeneous samples, we focus on those using individuals with non-specific LBP. Another review from Ruhe et al. (2011a) compares the center of pressure excursion of individuals with and without non-specific LBP only. This review provides a mere overview of differences across the parameters of postural sway. However, the proportion of significant and non-significant results on single parameters remains unclear.

The aim of this review is to identify the differences in postural control between individuals with and without non-specific LBP during quiet standing. To investigate these differences in neuromuscular and biomechanical parameters, we looked at case-control studies comparing individuals with and without non-specific LBP. Thereby, we set out to clarify relations between differences on both levels of motor control and identify superordinate strategies. The growing number of studies on the relation between LBP and the parameters of postural control provide essential information for an understanding the underlying reasons for LBP, not only at the biomechanical but also at the neuromuscular level. These results provide a basis for developing a diagnostic tool or an effective treatment in the future.

## Methods

This review has been devised along the guidelines of the PRISMA checklist (Liberati et al., 2009). However, it was not possible to conduct a meta-analysis due to the extent of missing values in the studies included.

### Search Strategy

**Figure 1:**
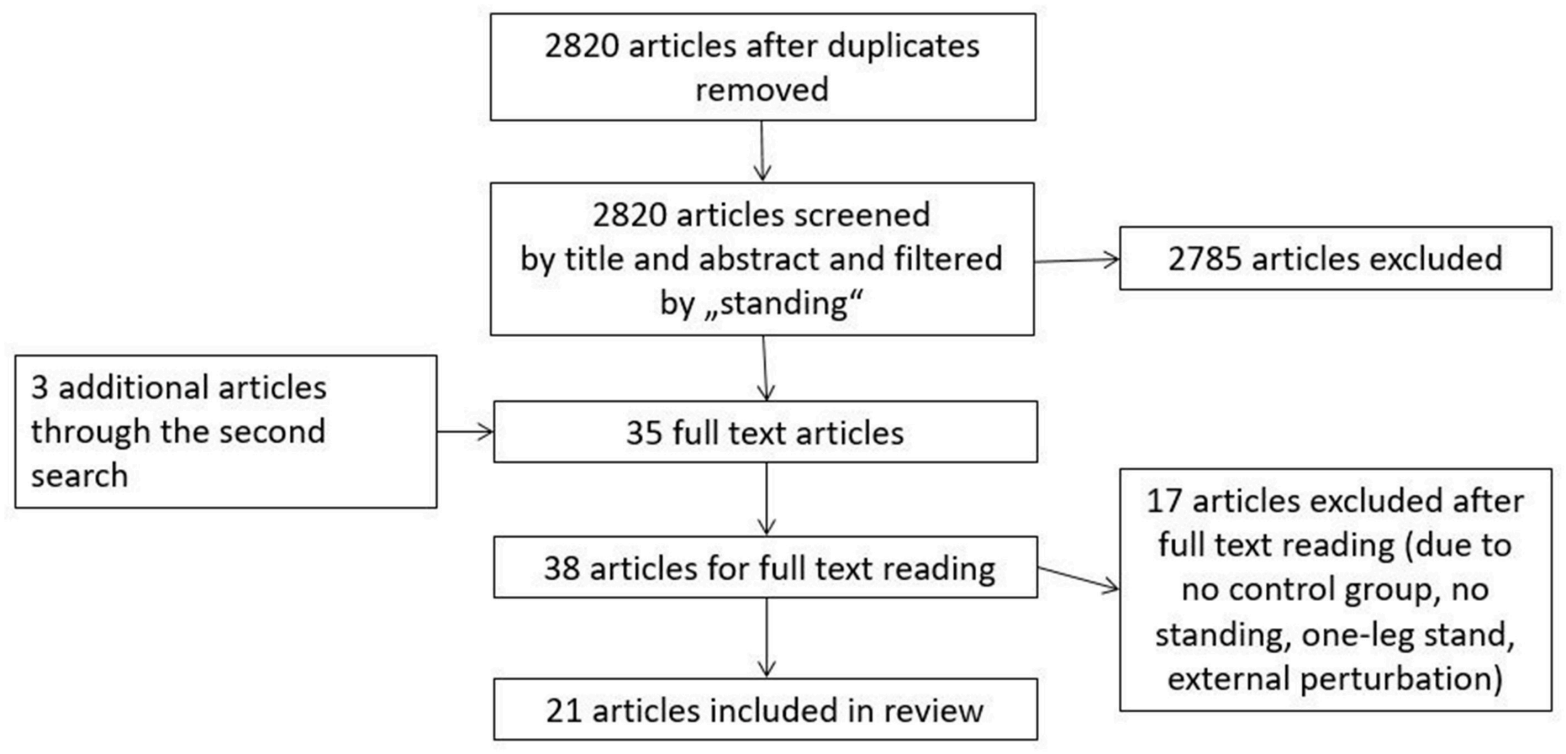
Flow chart of search strategy and study selection.

At first, six databases (Medline, SportDiscus, PsychInfo, PsychArticles, EMBASE, Scopus) were searched for articles published from 2000 to January 2018 using the following key words as a subject term search: “Low back pain” OR “lumbar pain” AND (“motor control” OR “coordination” OR “movement disorder” OR “variability” OR “stability” OR “proprioception” OR “muscle activation” OR “electromyography” OR “kinematics” OR “center of pressure” OR “range of motion” OR “muscle activity”) NOT (“invasive” OR “spinal stenosis” OR “injury” OR “case study” OR “disc herniation” OR “fractures” OR “amputation” OR “taping” OR “strength” OR “metabolic”). To extend the literature search, a second search was performed by using the key words “low back pain” OR “lumbar pain” in combination with “standing.” Lastly, the reference lists of the found articles were scanned for additional relevant studies. The search strategy was limited to articles written in English and German. The search strategy is presented in Figure 1.

### Study Selection

Eligible studies were screened by title and abstract to match inclusion and exclusion criteria. Articles were included if they studied persons with non-specific LBP and compared them with healthy controls with regard to any biomechanical and neuromuscular parameter of postural control while standing without movement. Biomechanical and neuromuscular parameters are used to quantify and measure motor control (Schmidt and Lee, 1999). Postural control is commonly quantified by looking at parameters like joint angles or CoP displacement. Electromyography measurements are typically used to determine the underlying activity of the neuromuscular system. The exclusion criteria used in our analysis were: Perturbation through external forces, study population of professional athletes, physiotherapeutic interventions, psychological interventions, specific back pain (e.g., with a diagnosis of herniated disk or back pain caused by injuries, pregnancy, or amputation), operation, evaluating medicine, studies without control group, studies about quality criteria of diagnostic systems and other reviews.

### Methodological Quality

A modified version of the Newcastle-Ottawa Scale (NOS) (Wells et al., 2000), which is a common quality assessment tool in case-control studies, was used to evaluate study quality. Deeks et al. (2003) found NOS to be an effective and feasible tool in the evaluation of quality in case-control studies. Based on this review, we added three questions from the Quality Assessment Tool for Quantitative Studies of the Effective Public Health Practice Project (Thomas, 2003) about the methods of data acquisition and adapted two other questions for our purposes (see Supplementary Data Sheet). The assessment tool consisted of 10 questions with a maximal score of 12 points with eight questions counting one point each and two questions being worth two points. The questions were divided into three categories: (1) selection of cases and controls, (2) comparability, and (3) data acquisition. The ten criteria for the assessment of the methodological quality were rated as being positive (“yes”), negative (“no”), or unknown (“not reported”), while only positively-rated criteria were awarded points. The maximum score was 12 points while eight questions counted one point each and two questions being worth two points. The quality assessment was performed independently by two raters. Differences were discussed and disparities were resolved, when necessary, with the help of a third person.

The assessment of evidence was modified based on van Tulder et al. (2003). Since there were no randomized controlled (RCT) or controlled studies (CT), we used high quality studies as RCTs and studies of moderate quality as CTs. Furthermore, we added “tendency” as a category for those cases, in which a slight majority of studies (between 50 and 75%) with higher average quality had reported the homogenous results.

### Data Extraction

Data extraction was performed independently by two raters. The variables considered were, study purpose and population, inclusion/exclusion criteria, conditions, equipment, outcome measures and results, which were extracted from the full-text version of the articles.

## Results

### Literature Search

After the removal of duplicates, a total of 2,820 articles was identified in the first search (see Figure 1). Next, these articles were scanned for appropriate fit by screening title and abstract according to the inclusion and exclusion criteria and with regard to the content of “standing.” This strategy was used to ensure that suitable articles were not rejected due to the great variety of search terms. In the second search, we identified three more relevant articles in the same databases. Upon further inspection, eighteen articles were rejected because they were about the investigation of external perturbations or 1-leg-stance, lacked a control group or did not perform a standing task. As a result, a total of 21 articles on back pain and postural control during quiet standing was included in this systematic review.

### Classification of Studies

The studies included investigated 1967 subjects (LBP: 1,158, asymptomatic: 809). All participants were 18 years or older. Testing in all studies was performed in a laboratory. The sample size of the studies varied from 8 (Nelson-Wong and Callaghan, 2010a) to 570 (Paalanne et al., 2008) participants, who experienced back pain at least at one point of testing (Paalanne et al., 2008) up to chronic pain with recurrent episodes for more than 18 months (Mok et al., 2004). There were seven studies (Gregory and Callaghan, 2008; Nelson-Wong et al., 2008; Nelson-Wong and Callaghan, 2010a,b; Gallagher et al., 2011; Gallagher and Callaghan, 2015, 2016), that assigned participants into case and control groups after testing. If pain developed in the course of standing, participants were assigned to the group of pain developer. Furthermore, there was one longitudinal study included (Paalanne et al., 2008). Further details of study characteristics are described in Table S1. To quantify the severity of pain, 10 studies used the Visual Analog Scale (VAS) (Mok et al., 2004; Brumagne et al., 2008; Nelson-Wong et al., 2008; Lafond et al., 2009; Nelson-Wong and Callaghan, 2010a,b; Gallagher et al., 2011; Sherafat et al., 2014; Gallagher and Callaghan, 2015, 2016) and three studies used the Numeric Rating Scale (NRS) (Claeys et al., 2011; Ruhe et al., 2011b; Kiers et al., 2015). The Oswestry Disability Index (ODI) or a modified version thereof (nine times; Brumagne et al., 2008; Lafond et al., 2009; Claeys et al., 2011, 2015; Johanson et al., 2011; Sherafat et al., 2014; Kiers et al., 2015; Ringheim et al., 2015; Schelldorfer et al., 2015) and the Roland-Morris Questionnaire (one time; Mok et al., 2004) were used to classify disability.

Various parameters were calculated from motion analysis data, CoP data, and electromyography (EMG) data. From kinematic data of 3D motion capture systems, joint angles were calculated. For CoP data, root mean square of CoP displacement, patterns of CoP displacement like drifts, mean CoP velocity, and frequency of CoP displacement were typically analyzed in the studies. CoP is referred to as the point at which the force vector of pressure of the body would start. It is used as a measure of the activity of the motor system in moving the COP, since it reflects for example the active anticipatory search process, or the output of a control process to maintain postural control via activity of the ankle musculature (Ruhe et al., 2011a).

Calculation of the different parameters differed in the studies. The root mean square of CoP displacement was calculated over different standing durations from 60 s (Ringheim et al., 2015) up to 90 s (Ruhe et al., 2011b). For CoP fidgets, defined as a quick and large displacement and a return of CoP to roughly the same position (Gallagher et al., 2011), frequency was calculated over 15 min of standing. Another parameter is CoP drift, defined as a slow continuous displacement of the average position of the CoP (linear or non-linear) (Gallagher et al., 2011). For drifting patterns, the average amplitude was calculated over 15 min of standing. The frequency content of CoP displacement was also analyzed by performing a Fourier transformation (Lafond et al., 2009; Kiers et al., 2015). Mean CoP velocity was calculates over standing durations differing from 60 s (Kiers et al., 2015; Ringheim et al., 2015) to 90 s (Ruhe et al., 2011b) in the different studies.

Body weight shifts were also used to examine differences between groups. They were defined as a weight transfer of more than 30% of one's body weight from one leg to another (Gallagher et al., 2011; Gallagher and Callaghan, 2015).

For EMG data root mean square of EMG activity over a certain time span and cross correlations between the right and left M. gluteus medii (GM) were the main parameters that were examined. Data acquisition and filter differed in every study. See Table S2 for an overview of data acquisition and processing methods for the EMG data.

In most studies, quiet standing was performed; however, some studies used challenging conditions, such as blindfolding in eight studies (Mok et al., 2004; Paalanne et al., 2008; Mazaheri et al., 2010; Johanson et al., 2011; Sherafat et al., 2014; Kiers et al., 2015; Ringheim et al., 2015; Schelldorfer et al., 2015), standing on perturbed surface in eight studies, or unstable surfaces in five studies (Claeys et al., 2011, 2015; Mazaheri et al., 2013; Kiers et al., 2015; Schelldorfer et al., 2015).

### Quality of Studies

Studies could achieve a maximum of 12 points in the quality score. As shown in Table S3, eight points is the highest quality score achieved by one study included (Mok et al., 2004). Additionally, eleven articles (Brumagne et al., 2008; Nelson-Wong et al., 2008; Paalanne et al., 2008; Lafond et al., 2009; Mazaheri et al., 2010; Claeys et al., 2011; Johanson et al., 2011; Ruhe et al., 2011b; Sherafat et al., 2014; Kiers et al., 2015; Schelldorfer et al., 2015) scored six or seven points. Nine papers (Brumagne et al., 2004; Gregory and Callaghan, 2008; Nelson-Wong and Callaghan, 2010a,b; Gallagher et al., 2011; Claeys et al., 2015; Gallagher and Callaghan, 2015, 2016; Ringheim et al., 2015) showed limitations in quality. For example, they did not use a matched group design or define their sample clearly. It is striking that two criteria were not reported in any of the studies. That is, none of the studies show whether outcome assessors were aware of the exposure status of the participants (question eight). A non-response rate was not reported in any of the studies either (question ten).

### Synthesis of Results

Operationalization in the studies on parameters of postural control differed. To provide an overview results are organized in subcategories of biomechanical and neuromuscular data. Because most of the studies on biomechanics CoP data was analyzed, we present these results in a separate section. At the beginning of each paragraph, we present the results. Afterward, confounding variables like participants' age, actual pain intensity, and chronicity were taken into consideration to find out the reasons for inconsistency. We additionally looked at differences in study quality.

#### Biomechanics During Standing

##### Kinematic data during standing

Position angles of spine and pelvis during standing represent one of the parameters investigated. Three studies (Brumagne et al., 2008; Claeys et al., 2015; Gallagher and Callaghan, 2016) did not find differences in position angles between individuals with and without LBP. All the study participants were between 22.8 years (Brumagne et al., 2008) and 33.5 years (Claeys et al., 2015). In two of the studies (Claeys et al., 2015; Gallagher and Callaghan, 2016) individuals were allocated to the LBP group as a result of their pain evaluation on a rating scale. Only one study (Brumagne et al., 2008) investigated persons with chronic pain, which lasted for at least 6 months. Based on consistent results, there is moderate evidence that position angles do not differ between subjects with and without LBP even though there is no high quality study.

##### CoP displacement during standing

There were 11 studies (Brumagne et al., 2004, 2008; Mok et al., 2004; Lafond et al., 2009; Mazaheri et al., 2010; Claeys et al., 2011, 2015; Johanson et al., 2011; Kiers et al., 2015; Ringheim et al., 2015; Schelldorfer et al., 2015) that investigated center of pressure (CoP) displacement. Nine of these studies asked participants to stand on a stable surface with their eyes open. However, their results were inconsistent. The majority of studies (Mok et al., 2004; Brumagne et al., 2008; Mazaheri et al., 2010; Claeys et al., 2015; Kiers et al., 2015) could not find a difference between groups. One of these studies, rated as being of high quality, found no difference (Mok et al., 2004). However, there is one study reporting no significant difference in CoP displacement between subjects with and without LBP (Kiers et al., 2015). Of the four studies showing a difference between the LBP and control group, two (Lafond et al., 2009; Claeys et al., 2011) reported smaller oscillations of the CoP, whereas another two studies (Brumagne et al., 2004; Ringheim et al., 2015) reported higher CoP displacement for patients with LBP as compared to controls. The age and number of participants as well as the intensity and duration of pain did not differ between those studies finding a difference between groups and those, that did not. Considering the slight majority in addition to the higher quality of studies reporting no difference between groups, we conclude, there is conflicting evidence with a tendency toward no difference in CoP displacement.

Seven of the eleven studies considered more challenging conditions such as standing on an unstable surface or with eyes closed. Among these seven studies, five (Brumagne et al., 2004, 2008; Claeys et al., 2011; Johanson et al., 2011; Schelldorfer et al., 2015) reported higher CoP displacement for the LBP group. Two (Claeys et al., 2011; Johanson et al., 2011) reported differences between standing on a foam and solid ground, three (Brumagne et al., 2004, 2008; Schelldorfer et al., 2015) when visual information was excluded. Two other studies did not find a difference between groups, one of which (Claeys et al., 2015) tested while standing on a foam ground while the other one (Ringheim et al., 2015) occluded visual information during testing. In most of the studies considering CoP displacement during more challenging conditions, the inclusion criterion for LBP required a pain duration of at least 6 months, however, information about actual length of the pain duration was missing. Thus, slightly different test protocols and different data processing do not seem to provide explanations for the inconsistency of results. A majority of studies found higher CoP displacement in more challenging conditions. Quality was also slightly higher in studies reporting higher CoP displacement in persons with LBP compared to controls. Taken together, the results for more difficult conditions are inconsistent, yet, there is a tendency for CoP displacement to be higher for persons with LBP than for controls.

Certain sway patterns, including fidgets and drifts, were also analyzed. Amongst three studies investigating fidget frequency, two studies (Gallagher et al., 2011; Gallagher and Callaghan, 2015) did not find a difference in fidget frequency between groups. Another study (Kiers et al., 2015) indicated higher fidget frequency for the LBP group. Both articles that found no difference investigated persons that did not have a history of LBP but developed pain while standing for 2 h. The study that found a difference (Kiers et al., 2015) examined persons who had an average pain duration of more than 3 years. It is possible that those results that are initially found to be inconsistent have a greater likelihood of being significant when pain is chronic and when it has a higher intensity at the time of testing. Finally, the small number of studies and the lack of a high quality study make it difficult to draw reliable conclusions.

Similarly, there are no clear results on the amplitude of sway patterns. Two studies examined the amplitude of drifting patterns (Lafond et al., 2009; Gallagher et al., 2011). One study (Lafond et al., 2009) reported a lower amplitude in LBP group compared to controls and another study (Gallagher et al., 2011) a lower amplitude in only men who develop pain during standing. The study that found a lower amplitude of sway patterns (Lafond et al., 2009) for the LBP group tested persons with chronic pain, while the second study (Gallagher et al., 2011) examined persons who had no history of LBP before testing. Therefore, there seems to be a tendency for lower amplitude in sway pattern with LBP.

Additionally, two articles investigated the frequency of CoP patterns. One (Kiers et al., 2015) found a higher frequency content for CoP sway on foam, which is less consistent, in persons with actual average pain for more than 3 years. This result means that the structure of their CoP displacement had a lager frequency range. However, they did not find any difference on stable surface. A separate study (Lafond et al., 2009) investigated CoP frequency content in persons with back pain compared to healthy controls on stable surfaces and showed lower CoP sway frequency content for the LBP group in the anterior-posterior direction as well as in the medio-lateral direction. Study quality and average age of participants are similar in the two studies. Owing to the small number of studies investigating this parameter and their varying results, further studies are required for reliable conclusions.

Another parameter observed in five studies was mean CoP velocity (Mok et al., 2004; Lafond et al., 2009; Ruhe et al., 2011b; Kiers et al., 2015; Ringheim et al., 2015). Two studies found lower CoP speed for the LBP group compared to the control group. One of these studies (Mok et al., 2004) investigated different vision conditions (eyes open vs. dimmed light vs. eyes closed) and another one (Lafond et al., 2009) confirmed the results with eyes open during prolonged standing. The study investigating different vision conditions has high quality (Mok et al., 2004). Another two studies found higher CoP speed in the LBP group compared to the control group: one with vision occluded (Ringheim et al., 2015) and the second examined the influence of the pain rating on sway velocity with eyes closed (Ruhe et al., 2011b). They found that sway velocity increased linearly with increasing pain intensity >4 on an NRS scale. Kiers et al. (2015) did not find a difference in CoP velocity between individuals with and without LBP on rigid surface and on foam. All studies investigated patients with chronic back pain. The average age of the LBP patients did not differ between studies. Inconsistency of results may be caused by different test protocols and different data acquisitions. Pain intensity in studies finding lower CoP sway velocity in LBP was quite low with <3. But in the study in which no difference was found, it was moderate with 4.5 on average. Further studies are necessary to draw more assertive and reliable conclusions.

Three studies working with muscle vibration (Claeys et al., 2011, 2015; Johanson et al., 2011) examined differences in the relative proprioceptive weighting, which is the ratio of dependence of proprioception between lumbar multifidus and triceps surae. All of these studies showed a higher dependence on ankle proprioception in LBP and less reliance on proprioception of low back muscles. The average age of participants was fairly young, and average pain intensity at the time of testing was mild. Pain duration differed between studies. All experiments were carried out from the same work group. Owing to the consistency of results, there is moderate evidence for a higher dependence on ankle strategy in persons with LBP compared to the controls.

There were some other CoP parameters that were assessed in single experiments only. Those were, for example, recurrence plots of standing position in combination with and without a cognitive task (Mazaheri et al., 2010) as well as stability index with and without a cognitive task (Sherafat et al., 2014). We do not report these results as these parameters provide only little indication for differences between groups,

##### Body weight transfers

Similarly divergent results exist for body weight transfers, which were investigated in three studies (Lafond et al., 2009; Gallagher et al., 2011; Gallagher and Callaghan, 2015). Gallagher et al. (2011) could not find a difference between groups. However, another study (Gallagher and Callaghan, 2015) found more body weight transfers in the first 15 min of standing for subjects who did not develop pain during prolonged standing. A third study (Lafond et al., 2009) found a lower number of shifting patterns for LBP group in the first 15 min of prolonged standing. This study investigated persons with chronic pain. Participants were approximately the same age [24.4 years (Gallagher et al., 2011) and 23 years (Gallagher and Callaghan, 2015)] in the first two studies. In the third study (Lafond et al., 2009), participants were 40 years on average. Due to the small number of studies and the lack of high quality studies, further studies are required to draw reliable conclusions about shifting patterns during standing.

#### Muscle Activity During Standing

Concerning the EMG data, there were large differences in the way the data were acquired and processed between the four studies. Nonetheless, the results are organized into sections in accordance with study parameters. One study (Ringheim et al., 2015) compared EMG activity of trunk muscles during standing. A higher erector spinae (ES) activation level at the start of a prolonged standing period in the persons with chronic recurrent LBP could be shown (Ringheim et al., 2015). Since there is only one study investigating this parameter further studies are required to confirm this finding.

Another parameter used to investigate differences between groups are cross-correlations of activity between two muscles to determine co-activation of the muscles. This parameter was examined in three studies. Two of the studies (Nelson-Wong et al., 2008; Nelson-Wong and Callaghan, 2010b) showed cross-correlations between the left and right GM. The authors attribute this finding to a higher bilateral co-activation of the GM in persons with LBP compared to the control group. In these studies, persons with LBP developed their pain during a period of prolonged standing, but did not have pain before testing. The third study (Nelson-Wong and Callaghan, 2010a), which had a similar sample, only found differences when the surface was sloped. All experiments were performed by the same working group. Therefore, these results provide an indication for higher bilateral co-activation of GM in persons who developed back pain during prolonged standing.

## Discussion

The objective of this review is to provide an overview of the differences in postural control on biomechanical and neuromuscular level between persons with and without non-specific LBP during quiet standing. The majority of the studies included shows moderate quality while only few studies of high quality could be found. Overall, we found an indication for a stiffen lumbar pelvic area through increased activity of hip and back muscles. Technical procedures and the operationalization of parameters differed in every study. Nonetheless we decided to group results into different sections. In the following, we discuss our results on position angles, CoP displacement, relative proprioceptive weighting and EMG activity.

In the studies we reviewed, we did not find any evidence for differences between individuals with and without non-specific back pain with regard to joint angles. Therefore, our results are in line with those from the review of Laird et al. (2014) even though we focused on studies investigating patients with a specific reason for pain. Due to the confirmation of these results, we conclude that there is no evidence for differences between individuals with and without non-specific LBP. One possible reason for this result might be that the anatomical variety of hip, pelvis, and spine between the individuals is too wide as Laird et al. (2014) point out.

Regarding CoP in standing, in the reviewed studies we also found no difference between groups in CoP sway in standing on a stable surface with eyes open. However, we found an indication for differences between groups in situations with higher postural demands. In terms of standing on a stable surface with eyes open, the majority of the studies included in our review could not find any difference between groups. In the condition where visual information is excluded, a slight majority of studies included in our review showed a higher CoP sway for the LBP group. For that reason and owing to the higher quality of studies reporting a difference, we conclude that there is a tendency for a higher CoP sway in LBP group during standing conditions with higher demands. These results are somewhat different from the results of Mazaheri et al. (2013) and Ruhe et al. (2011a). Regarding standing on a stable surface our results are in line with those of Mazaheri et al. (2013), who did not find any differences, but not with Ruhe et al. (2011a), who reported differences in postural sway. In their review Ruhe et al. (2011a) only referred to studies confirming differences in parameters, while the ratio to studies with non-significant differences in single parameters is not mentioned. In contrast, when evaluating a parameter, we also considered non-significant results, since it is also necessary to see how consistent the results were. In terms of standing on a stable surface with vision occluded our results are in line with Ruhe et al. (2011a), who show a gain of statistical differences for postural sway in conditions with occluded vision, but not with Mazaheri et al. (2013), who could not find any differences. Yet, in their review Mazaheri et al. (2013) did not specifically look at non-specific LBP, which may be the reason for their differing results, while Ruhe et al. (2011a) focused on patients with non-specific LBP, as we did in the present review. The significant differences could be explained by a reduced proprioception in persons with LBP (Laird et al., 2014). An impaired sensory input from muscles and joints through pain has a lager impact with closed eyes. When vision is occluded, visual information cannot be used to compensate impairments in proprioception. The integration of multiple sensory information, which includes proprioceptive, vestibular, and visual systems, is necessary for balance as well as spatial orientation. Visual information seems to be especially important in the control of quiet stance (Gill et al., 2001). However, it seems that when vision is occluded deficits in sensory inputs cannot be compensated. Further studies, which do not consider quiet standing but perturbation through external forces (Henry et al., 2006; Götze et al., 2015) confirm that CoP displacement is higher in individuals with non-specific LBP compared to controls for situations with higher demands.

Our results on the proprioception strategy match those of previous studies. We found moderate evidence for a different proprioception strategy in persons with non-specific LBP, which is demonstrated by a higher dependency on ankle strategy in persons with LBP (Claeys et al., 2011, 2015; Johanson et al., 2011). A higher dependency on ankle strategy means, that balance is mainly regulated through ankle movement, whereas the ability to regulate balance through hip and lumbar motion is restricted. In hip strategy, CoP is rearranged by flexion and extension of the hip joint. It is reported that mainly ankle strategy is used during quiet standing (Horak and Nashner, 1986). In individuals with LBP, for which there was some evidence of an impairment in postural control (Claeys et al., 2011; Johanson et al., 2011), differences would only become clear with tasks where a hip strategy is necessary to balance the CoP, like in tasks with higher demands. For example, Götze et al. (2015) and Henry et al. (2006) showed differences between groups when standing was perturbated. Therefore, especially in tasks with higher demands, hip movements seem to be important for balance control (Horak and Nashner, 1986).

Taken together, the findings for CoP and the dependency on different postural control strategies suggest that the ability to regulate balance through hip and lumbar motion is restricted in more difficult tasks during standing. According to the model of Hodges and Tucker (2011) the prohibition of hip and trunk flexion in tasks with higher demands may be a protective strategy, aimed at avoiding the pain felt in the respective area. However, we cannot confirm any effects of pain for quiet standing on solid ground with eyes open. Kiemel et al. (2011) postulate, that one goal of the postural control is to stabilize with minimal muscular activity. Accordingly, as long as the CoP is within the base of support, there is no need to produce more muscular activity. Additional activity would only mean a higher energy expenditure. Thus, during quiet standing when balance is not at risk, there is no need to minimize CoP sway (Kiemel et al., 2011).

Furthermore, there is an indication for differences in co-activation of the GM and activation of ES for the results of the EMG data. For patients with LBP, there seems to be a trend toward higher co-activation of GM and higher ES activity. A generally higher activity in hip and spine muscles during quiet standing can be a strategy to counter spinal instability (Ghamkhar and Kahlaee, 2015) since one function of the GM and the ES is to stabilize the pelvis. Therefore, forces acting on the lumbar spine are reduced. Since both muscle groups act against flexion of hip and spine, flexion seems to be prohibited. Thus, the use of hip strategy is impaired. As Hodges and Tucker (2011) propose in their model, we do have an indication for an altered neuromuscular activity in LBP.

If we consider that there is a relationship between the activity of the neuromuscular system and the resulting biomechanics, combining the results of all parameters helps one to obtain a full impression of the differences between groups. A higher reliance on ankle strategy for maintaining balance seems to be the consequence of impaired hip flexion through a higher co-activation of GM in subjects with LBP. Since hip flexion and extensions play a role in stabilizing the CoP, an impaired hip flexion can result in higher excursions of CoP in more challenging conditions. Hip strategy is suggested to be used, especially if there are large and strong disturbances to the equilibrium (Shumway-Cook and Woollacott, 2007). Therefore, when standing on an unstable surface, where the disturbance of the equilibrium is larger, differences become more apparent.

Nonetheless, there are limitations to our findings. Participants age, definition of cases and controls, pain duration as well as pain intensity differed between studies. By considering the selection of cases and controls in the quality assessment as well as the mentioning the comparability of pain intensity and duration as main confounders, we tried to specify our results. The results of comparisons are limited by different survey methods when it comes to testing situation, data acquisition, and data. For instance, instructions for the participants differed between studies. In some studies, participants were asked to stand as quietly as possible, whereas in other studies they were instructed to stand in a relaxed manner. Another example of differences in the survey methods was the standardization of the feet position. Different stand widths were used and there is no recommendation for the feet position by which to get the most reliable data (Ruhe et al., 2010). To allow better comparison, a standardization is necessary. Standardized procedures would help to clarify the relationships between non-specific LBP and postural control. They can also help to identify a differential effect through systematic variations. Besides, another limitation is that experimental settings may differ from standing in a natural environment because people probably focus more on the way they stand when they are being observed than when they stand or walk freely in their everyday life. As a consequence, without employing such standardizations, the effects found in these studies should be interpreted with caution. Nonetheless, we found some tendencies for reliable effects across the disparate methodological procedures used in the studies we examined based on the current literature.

Furthermore, it is not clear whether CoP displacement is the best parameter for an investigation of postural control. The results of recent studies in which the amount of joints involved in balance control was examined suggest that models with up to seven degrees of freedom had the highest shared variance with CoP (Kilby et al., 2015). Stabilization of postural balance is, thus, reached through a complex pattern of joint motion, which is why hip motion plays only a marginal role. However, CoP is a parameter that is used in many studies to measure postural control.

Another controversial point is the methodology of the quality assessment. We used the highly recommended instrument, NOS, in the assessment of studies but the reporting on key criteria was incomplete and some criteria were not mentioned in any of the studies at all. Therefore, NOS seems to utilize criteria that are closer to an ideal rather than a realistic conception and very difficult, if not impossible, to fulfill. Thus, we suggest that the standards of describing the methodological approach have to be modified. In our evaluation of study quality, we made conservative estimates of quality, namely, we awarded no points when the criteria were not reported or not fulfilled. Consequently, the methodological quality might be underestimated because of insufficient reporting rather than poor study design or poor methodological approach.

The results of this review imply that there seem to be differences in postural control between individuals with and without non-specific back pain in quiet standing, which become more evident in situations with high demands. As we pointed out for CoP displacement, there is an indication for differences in standing situations where individuals with LBP have to adapt to perturbation through external forces like when standing surface is translated. Since the results are based on the existing studies, which used different acquisition and processing procedures, the results have to be verified by new studies. It is necessary to replicate the results of the reviewed studies with standardized procedures in order to draw more reliable conclusions on the differences in postural control during standing. Furthermore, it might be interesting in the future to look for differences between individuals with and without LBP when the standing task is even more demanding, since during a large CoP displacement, which occurs when standing is perturbed, the role of hip and lumbar region plays an even greater role for postural control.

## Conclusion

In sum, we conclude that persons with and without non-specific LBP differ in postural control concerning certain parameters. Even though there is no strong evidence for any of the parameters alone, findings match if we combine results of direct measurements of neuromuscular system via EMG and measurements of the resultant biomechanics. Altered postural control is identified in persons with non-specific back pain. It appears in an impaired flexion in the hip-pelvis region through higher baseline activation of hip and trunk muscles, more ankle strategy for balance control and higher CoP sways under more difficult tasks. To draw more definite conclusions on the differences in postural control during standing, it is necessary to replicate the results of these studies with standardized procedures since these findings were based on studies which utilized different methodological procedures.

## Author Contributions

CK and FH have made substantial contributions to the conception, analysis and interpretation of data, and drafted and revised the manuscript critically for important content.

### Conflict of Interest Statement

The authors declare that the research was conducted in the absence of any commercial or financial relationships that could be construed as a potential conflict of interest.
