# Supplementary material for: Non-specific Low Back Pain and Postural Control During Quiet Standing—A Systematic Review
Source: Front Psychol. 2019 Mar 22;10:586. doi: 10.3389/fpsyg.2019.00586 (PMC6440285; doi:10.3389/fpsyg.2019.00586)
Supplement: Supplementary file 1 [file Table_1.DOCX]

Supplementary Material

Non-specific low back pain and motor control during quiet standing - A systematic review

Cathrin Koch*, Frank Hänsel

*** Correspondence:** Cathrin Koch: koch@sport.tu-darmstadt.de

## Supplementary Tables

Table 1: Data extraction of included studies

| Author (year) | Study population | Conditions | Outcome measures | Main results |
| --- | --- | --- | --- | --- |
| Brumagne, Cordo (39) | 20 LBP (10 young, mean age = 25 years;  and 10 older LBP mean age = 63 years;  mean age = 63 years, 20 controls (10 young and 10 older), matched according to age | 4 conditions in standing still: 1. control condition, 2. bilateral vibration on triceps surae tendons, 3. bilateral vibration of the tibialis anterior tendons, 4. bilateral vibration of the paraspinal muscle bellies;  60 sec per trial, vibration after 15 sec. for 15 sec. | CoP before and after vibration,  RMS CoP,  postural recovery times | larger RMS CoP in anterior-posterior direction for LBP group; with bilateral triceps surae vibration LBP and older adults had larger CoP displacements; with vibration on paraspinal muscles, the displacement was larger in control group and young adults |
| Brumagne, Janssens (27) | 56 LBP (22.7 years),  22 controls (23.2 years), matched according to age | 1. stable surface, eyes open; 2. stable surface blindfold; 3. vision occlusion, ballistic arm movement; 4. vision occlusion, short soleus vibration; 5. vision occluded long soleus vibration | body inclination, CoP position, RMS | no difference in body inclination; LBP CoP position more ventral on stable surface and anticipated postural instability |
| Claeys, Brumagne (31) | 106 LBP (18.5 years), 50 controls (19.6 years),  age-matched | 8 trials: 4 on stable surface, 4 on foam; normal, with arm movement, vibration triceps surae, vibration lumbar multifiduus | RMS CoP, relative proprioceptive weighting | NSLBP less sway on stable surface (trial 1); NSLBP more sway on foam (trial 5); NSLBP more sway in backward direction with vibration on triceps surae (trial 3); NSLBP higher values for RPW in both surfaces |
| Claeys, Dankaerts (33) | 43 LBP (19.1 years), 61 controls (19.2 years), matched according to age | Condition 1: stable support surface (1) quiet standing, (2) quiet standing, ballistic shoulder flexion at 30s, (3) quiet standing, bilateral triceps surae muscles vibration, (4) quiet standing, bilateral lumbar multifidus muscles vibration; Condition 2: unstable support surface (foam) (5) quiet standing, (6) quiet standing, ballistic shoulder flexion at 30s, (7) quiet standing, bilateral triceps surae muscles vibration, (8) quiet standing, bilateral lumbar multifidus muscles vibration | RMS CoP, relative proprioceptive weighting ratio (RPW), five sagittal angles | no significant difference between groups in the RMS scores of the COP displacements; difference between groups in the muscle vibration trials was shown: the No LBP–LBP group showed more reliance on ankle proprioceptive inputs in the stable standing; on the unstable support surface, the No LBP–LBP group showed significantly higher RPW values compared to the No LBP–No LBP group; no difference in postural angles |
|  |  |  |  |  |
|  |  |  |  |  |
|  |  |  |  |  |
| Gallagher, Nelson-Wong (21) | 13 PD, 27 NPD,  average age 24.4 ± 2.9 years | 2h standing on even surface, occupational simulation | body weight shifts,  drifts,  fidget (frequency, amplitude, number) | NPD and female PD were consistent for many of the variables; however, male PD did not show similar patterns to the other groups, especially for anterior–posterior (AP) shift amplitude and total body weight asymmetry |
| Gallagher and Callaghan (22) | 14 PD (23.0 years), 18 NPD (22.2 years) | 2h standing on even surface, occupational simulation | body weight shifts,  fidgets (frequency, magnitude),  lumbar spine movement | non-PD performed a higher frequency of lumbar spine flexion/extension fidgets and body weight transfers in the first 15 min |
| Gallagher and Callaghan (23) | 9 PD,  8 NPD at even surface;  23 years; age range 18­–35;  only three PD when surface was decreasing | 2 standing conditions: level and sloped (16°) ground; pretrials of 60 sec, then 75 min prolonged standing | joint angles (trunk, spine, hip, trunk-tight),  CoG,  lumbar spine fidgets | when standing on the sloped surface, the average maximum pain scores for PDs was 58% less compared to reports while standing on level ground, only three of nine still developed pain; non-PD performed more lumbar spine fidgets in the sagittal plane than PDs, independent of the condition |
| Gregory and Callaghan (24) | 13 PD,  3 NPD, 24.3 years | 2h prolonged standing on confined working space, occupational simulation | total number of gaps in activation,  average activation amplitudes, number of shifts in EMG,  mean flexion-extension,  lateral flexion and axial rotation, back extensor muscle oxygenation | three regression models for prediction of LBP were calculated, the best equation was with 15 of 16 subject: Low back discomfort = (0.062 number of shifts in CoP [AP]) + (0.113 number of gaps in LGM activation) + (1.678 degree of axial twist (8)) + (0.186) |
| Johanson, Brumagne (34) | 16 LBP (22.0 years), 16 controls (22.7 years) | 1.A. upright stance–stable support surface 1.A.1. without vision, 1.A.2. without vision, bilateral triceps surae muscle vibration, 1.A.3. without vision, bilateral lumbar multifidus muscle vibration 1.B. upright stance–unstable support surface (foam) 1.B.1. without vision, 1.B.2. without vision, bilateral triceps surae muscle vibration, 1.B.3. without vision, bilateral lumbar multifidus muscle vibration 2. back muscle fatigue (day 2) same conditions as day one but fatigued | COP anterior–posterior = Mx/Fz, RMS, RW (relative proprioceptive weighting TS/MF = absolute TS/(abs TS + abs MF) | higher RMS values on unstable surface in LBP compared to controls, on unstable surface LBP showed larger posterior sway with ankle vibration, but smaller anterior sway compared to controls when back muscle vibration was applied |
| Kiers, van Dieen (30) | 182 LBP NRS <2 (39 years),  33 LBP NRS > 3 (41.3 years) | (1) upright standing with transparent safety glasses and with eyes open, (2) upright standing, and (3) upright standing on a foam support surface (Airex balance pad, 6 cm thick), (4) vision occluded, solid surface, vibration paraspinal musculature, (5) vision occluded, solid surface, vibration triceps surae, (6) vision occluded, foam, vibration paraspinal musculature, (7) vision occluded, foam, vibration triceps surae | CoP: sway, velocity, frequency, regularity,  reaction to vibration: TS vibration, LPM vibration, proprioceptive weighting | Subjects with LBP had significantly higher factor scores for sway pattern, which contained higher frequencies and which was less regular; subjects with LBP showed a smaller response to TS vibration; when standing on a solid surface, recovery was significantly slower in subjects with LBP. |
| Lafond, Champagne (28) | 12 CLBP (41.5 years),  12 controls (40.0 years),  matched according to age and sex | 2 trials quiet standing before and after 30 min prolonged standing | CoP patterns,  mean CoP speed,  RMS CoP,  mean power frequency | less shifting and drifting in A-P direction in CLBP during prolonged standing; amplitude of CoP displacement—no difference; COP speed slower in CLBP in the M-L direction during prolonged standing; COP RMS in LBP less in the A-P direction; mean COP frequency less in LBP in the A-P and M-L directions |
| Mazaheri, Salavati (38) | 20 LBP (26.1 years), 20 controls (25.0 years) | (1) standing on a force platform with open eyes (rigid-open); (2) standing on a force platform with closed eyes (rigid-closed); and (3) standing on the foam with closed eyes (foam-closed); additionally, a backward digit span task was selected as the cognitive task | recurrence plots,  COP-displacement anteroposterior (AP) and mediolateral (ML),  quantified by RQA (recurrence quantification analysis) % recurrence, % determinism, entropy and trend,  postural performance | interactions between group and cognitive difficulty were shown for % recurrence, % determinism and trend in the AP direction. While healthy participants decreased % recurrence and trend by increasing the level of cognitive difficulty, the LBP patients did not |
| Mok, Brauer (20) | 24 CLBP (36.6 years),  24 controls (36.9 years),  matched according to age and sex | 12 trials: unilateral vs. bilateral stance; flat surface, 9cm; vision eyes open, dimmed light, eyes closed | number of successful trials, shear forces,  CoP displacement, CoP speed | no difference between groups in the amount of successful trials, shear forces and CoP displacement; LBP slower CoP velocity |
| Nelson-Wong, Gregory (25) | 15 PD, 8 NPD,  23.9 years | 2h prolonged standing on confined working space,occupational simulation | co-activation coefficient of 8 pairs of muscles | no main effect of time or main effect of group for co-activation pattern for all muscles, considering all subjects; but if only 17 subjects are included is there a main effect for the group; the predictive utility of examining a single factor of co-activation of the left and right GM muscles was relatively good, which 17/23 participants correctly predicted |
| Nelson-Wong and Callaghan (18) | 8 PD,  8 NPD, 22.2 years | 1.2h prolonged standing in confined working space, occupational simulation; 2. same on sloped surface | relative joint angles at the ankle, knee, hip, and trunk as well as the global pelvis angle;  co-contraction Indices (CCI) for all muscle pairs | PD showed lower pain score on sloped surface; non-significant interaction between standing condition and PD/NPD group for gluteus medius CCI |
| Nelson-Wong and Callaghan (26) | 20 PD (23.45 years), 23 NPD (22.13 years) | 2h prolonged standing in confined working space, occupational simulation | co-activation coefficient,  co-contraction index | PD had higher levels of muscle co-activation at the beginning compared to non LBD for flexor-extensor pairs; PD had increased co-activation muscle patterns as a precursor to the increase in their subjective reports of pain development |
| Paalanne, Korpelainen (19) | N = 874 (19 years);  analysis of 743 individuals; young adults born between 01.07.85 and 30.06.86 | standing with their feet together and arms down their sides, 4 times for 60 sec., twice with eyes open and twice with eyes closed | cluster analysis according to symptoms,  body sway area,  lifetime prevalence | no difference in trunk muscle strength and body sway between subjects in different clusters |
| Ringheim, Austein (35) | 17 CLBP (39.0 years), 21 controls (40.2 years) | 60 sec standing with vision occluded, 15 min quiet standing with eyes open, 60 sec standing with vision occluded | CoP RMS,  CoP speed,  muscle activation (% RMS max),  variability in muscle activation | CLBP in prolonged standing increased postural compared to HCs, reaching statistical significance for CoP speed and a trend for CoP area and A-P CoP RMS; relative muscle activation level at the start and during the prolonged standing was higher in CLBP for all muscles except for GM; no difference in CoP measures in quiet standing |
| Ruhe, Fejer (32) | 77 LBP (37.7 years), 77 controls (37.2 years) | 3 successful trials of 90 sec quiet standing | mean CoP velocity | with increasing pain rating CoP sway velocity, velocity increases linearly |
| Schelldorfer, Ernst (36) | 57 LBP (39.21 years),  22 controls (38.55 years) | 1. feet together, stable surface, eyes open; 2. feet together, stable surface, eyes closed; 3. feet together, on foam, eyes open | MADposition, MADvelocity | higher MAD position in condition 1 and 2 for LBP group in thoracal and lumbar spine in frontal plane; MADvel of t-spine lower in control group in frontal plane |
| Sherafat, Salavati (29) | 15 CLBP (23.07 years),  15 controls (23.20 years),  matched according to age, gender, height and weight | (1) single task, stability level 5, eyes open; (2) dual task, stability level 5, eyes open; (3) single task, stability level 5, eyes closed; (4) dual task, stability level, 5 eyes closed; (5) single task, stability level 3, eyes closed; and (6) dual task, stability level 3, eyes closed | anterior-posterior stability index (APSI),  medio-lateral stability index (MLSI),  overall stability index (OSI),  verbal reaction time | interaction of group by cognitive task difficulty was significant only in the stability level of 5, eyes-closed condition for APSI, MLSI, and OSI; with dual task condition, increase in stability indices in LBP |

CLBP chronic low back pain; PD pain developer; NPD non pain developer; MAD mean average deviation; RMS root mean square; ES m. erector spinae; RA m. rectus abdominis; GM m. gluteus medius; TrA m. transversus abdominis; IO m. obliquus internus; EO m. obliquus externus; DM deep m. mulitfiduus, SM superficial m. multifidus; TS m. triceps surae; LPM lumbar paraspinal musculature; TA m. tibialis anterior; GA m. gastrocnemius.
