# Supplementary material for: Non-specific Low Back Pain and Postural Control During Quiet Standing—A Systematic Review
Source: Front Psychol. 2019 Mar 22;10:586. doi: 10.3389/fpsyg.2019.00586 (PMC6440285; doi:10.3389/fpsyg.2019.00586)
Supplement: Supplementary file 2 [file Table_2.DOCX]

Supplementary Material

Non-specific low back pain and motor control during quiet standing - A systematic review

Cathrin Koch*, Frank Hänsel

*** Correspondence:** Cathrin Koch: [koch@sport.tu-darmstadt.de](mailto:koch@sport.tu-darmstadt.de)

**Table 2: Different data acquisition and processing methods for EMG data**

| **Study** | **Position of electrodes** | **Sampling frequency** | **Normalization procedure** | **Filter** |
| --- | --- | --- | --- | --- |
| Nelson-Wong & Callaghan (18) | Lumbar Erector Spinae  (above and below L1 spinous process)  and Gluteus Medius (1 inch distal to the midpoint of the iliac crest);  2 cm centre-to-centre  inter-electrode distance | 2048 Hz | Maximal voluntary contractions (MVCs) with manual resistance | dual-pass, 4th-order, zero-lag Butterworth, effective cutoff frequency of 400 Hz;  when heart rate contamination was observed the EMG was band-pass filtered with a dual-pass, 4th-order, zero-lag Butterworth, 35–400 Hz |
| Nelson-Wong et al. (25) | Lumbar Erector Spinae based on  the work of McGill (1991) and Gluteus medius electrodes  was 15 cm inferior and 5 cm posterior of each iliac  crest;  2 cm centre-to-centre inter-electrode distance | 2048 Hz | Maximal voluntary contractions (MVCs) with manual resistance;  Down sampled to 32 Hz prior to data analysis | No details provided;  Calculation of cross-correlations |
| Nelson-Wong & Callaghan (26) | Lumbar Erector Spinae  (above and below L1 spinous process)  and Gluteus Medius (1 inch distal to the midpoint of the iliac crest);  2 cm centre-to-centre inter-electrode distance | 2048 Hz | Maximal voluntary contractions (MVCs) with manual resistance | All EMG had any systematic bias removed  and was then low-pass filtered with dual-pass, 4th-order, zero  lag Butterworth, effective cutoff frequency of 400 Hz; when heart rate contamination was observed EMG was high-pass filtered with a dual-pass, 4th-order, zero lag Butterworth, 35 Hz; some EMG signals also contained 60 Hz electrical noise contamination,  which was removed with a band-stop filter (dual-pass, 4th-order, zero lag Butterworth, 59–61 Hz) |
| Ringheim et al. (35) | bilaterally from the erector spinae (ES), gluteus medius (GM)  2 cm centre-to-centre inter-electrode distance | 1500 Hz | MVC | low pass filtered with an 8th-order recursive Butterworth filter of 20 Hz and 500 Hz, respectively;  In order to remove artefacts resulting from electrocardiography and movement, a 40 Hz high-pass filter was used |
