# Supplementary material for: Non-specific Low Back Pain and Postural Control During Quiet Standing—A Systematic Review
Source: Front Psychol. 2019 Mar 22;10:586. doi: 10.3389/fpsyg.2019.00586 (PMC6440285; doi:10.3389/fpsyg.2019.00586)
Supplement: Supplementary file 3 [file Table_3.docx]

Supplementary Material

Non-specific low back pain and motor control during quiet standing - A systematic review

Cathrin Koch*, Frank Hänsel

*** Correspondence:** Cathrin Koch: [koch@sport.tu-darmstadt.de](mailto:koch@sport.tu-darmstadt.de)

**Table 3: Quality assessment of included studies**

| Study | 1 | 2 | 3 | 4 | 5 | 6 | 7 | 8 | 9 | 10 | Score |
| --- | --- | --- | --- | --- | --- | --- | --- | --- | --- | --- | --- |
| Brumagne, Cordo (39) | k | 0 | k | k | 1 | 1 | 1 | k | 1 | k | 4 |
| Brumagne, Janssens (27) | 2 | 0 | 1 | 0 | k | 1 | 1 | k | 1 | k | 6 |
| Claeys, Brumagne (31) | 2 | 0 | 1 | 0 | 1 | 1 | 1 | k | 1 | k | 7 |
| Claeys, Dankaerts (33) | 0 | 0 | 1 | 0 | k | 1 | 1 | k | 1 | k | 4 |
| Gallagher, Nelson-Wong (21) | 0 | 0 | 1 | 1 | k | 1 | 1 | k | 1 | k | 5 |
| Gallagher and Callaghan (22) | 0 | 0 | 1 | 1 | k | 1 | 1 | k | 1 | k | 5 |
| Gallagher and Callaghan (23) | 0 | 0 | k | 1 | k | 1 | 1 | k | 1 | k | 4 |
| Gregory and Callaghan (24) | 0 | 0 | 1 | 1 | k | 1 | 1 | k | 1 | k | 5 |
| Johanson, Brumagne (34) | 2 | 0 | 0 | 1 | k | 1 | 1 | k | 1 | k | 6 |
| Kiers, van Dieen (30) | 0 | 0 | 1 | 0 | 2 | 1 | 1 | k | 1 | k | 6 |
| Lafond, Champagne (28) | 1 | k | k | 0 | 2 | 1 | 1 | k | 1 | k | 6 |
| Mazaheri, Salavati (38) | 2 | 0 | 1 | k | k | 1 | 1 | k | 1 | k | 6 |
| Mok, Brauer (20) | 1 | 0 | 1 | 1 | 2 | 1 | 1 | k | 1 | k | 8 |
| Nelson-Wong, Gregory (25) | 0 | 0 | 1 | 1 | k | 1 | 1 | 1 | 1 | k | 6 |
| Nelson-Wong and Callaghan (18) | 0 | 0 | 1 | 1 | k | 1 | 1 | k | 1 | k | 5 |
| Nelson-Wong and Callaghan (26) | 0 | 0 | 1 | 1 | k | 1 | 1 | k | 1 | k | 5 |
| Paalanne, Korpelainen (19) | 1 | 1 | 1 | 1* | 1 | k | k | k | 1 | k | 6 |
| Ringheim, Austein (35) | 1 | 0 | k | 0 | k | 1 | 1 | k | 1 | k | 4 |
| Ruhe, Fejer (32) | 1 | 1 | 1 | 0 | 0 | 1 | 1 | k | 1 | k | 6 |
| Schelldorfer, Ernst (36) | 1 | 0 | 1 | 0 | 2 | 1 | 1 | k | 1 | k | 7 |
| Sherafat, Salavati (29) | 1 | 0 | 0 | 1 | 2 | 1 | 1 | k | 1 | k | 7 |

Questions: 1 Adequate case definition; 2 Representativeness of the cases; 3 Selection of controls; 4 Definition of controls; 5 Comparability of cases and controls; 6 Valid data acquisition; 7 Reliable data acquisition; 8 Blinding of investigators; 9 Same method of ascertainment for cases and controls; 10 Reporting of non-response rate; Evaluation: 1 “Yes”; 0 “No”; k not reported; 2 more than just standard; * Definition of control group is given, but in the analysis considered as one of six clusters
